# Core Self-Evaluation and Work Engagement: Moderated Mediation Model of Career Adaptability and Job Insecurity

**DOI:** 10.3389/fpsyg.2019.02093

**Published:** 2019-09-18

**Authors:** Kieun Yoo, Ki-Hak Lee

**Affiliations:** Department of Psychology, Yonsei University, Seoul, South Korea

**Keywords:** career adaptability, core self-evaluation, work engagement, job insecurity, career construction theory

## Abstract

This study examined the moderated mediation roles of job insecurity through career adaptability on the relation between core self-evaluation (CSE) and work engagement. A total of 335 Korean full-time employees who had been employed for <3 years responded to the questionnaire survey. Excluding missing data and outliers, data from 324 participants (men = 82, women = 242) were analyzed using SPSS 25.0 and SPSS PROCESS Macro. The results indicated that job insecurity moderated the indirect association between CSE and work engagement via career adaptability. This study further expanded the understanding of newly employed employees’ adaptation. Implications and limitations of the study and suggestions for further study were discussed.

## Introduction

As the nature of the work environment becomes globalized and diversified, organizations can no longer be responsible for individuals’ career development (Biemann et al., 2012). Therefore, individuals are taking on the responsibility for their own careers (Kim and Jyung, 2011). In the *status quo*, individuals are facing the difficulties of adapting to new job demands and changing environments (Savickas et al., 2009; Savickas and Porfeli, 2012; Yang et al., 2019).

In Korea, the high turnover and low retention rates of new employee are serious problems. According to the Korea Employers Federation (The Korea Employers Federation [KEF], 2016), the annual turnover rate of new hires increased by 5% point, marking 27.7% over the last 5 years. In other words, nearly one in three newly recruited employees will leave their employer within a year. This number indicates a great loss for both the individuals who have devoted their time and effort on the job search and the organizations that have invested their resources on recruiting (Lee, 2010), trained employees, and lost human resources who had internalized the organizational norms and values. The primary cause of the early turnover is the employees’ failure to adapt to the job and the organization (49.0%) (The Korea Employers Federation [KEF], 2016). Thus, new employees’ adaptation is an important variable affecting individuals and organizations in Korea. The present study aimed to determine the internal adaptation mechanism of the newly employed and explore the organizational environment that may influence this process.

According to Kahn (1990), work engagement is defined as the expression and utilization of the preferred self, connecting the individual’s cognition, emotion, and behavior to active and satisfying performance. Therefore, work engagement is defined that workers input their physical, cognitive, and emotional energy in the process of performing their job (Kahn, 1990). Based on the initial work of Kahn (1990), Schaufeli et al. (2002) defined work engagement as a positive, enthusiastic, work-related state of mind that is composed of three sub-factors: vigor, dedication, and absorption (Schaufeli et al., 2002). Work engagement is considered a positive predictor of career adaptation (Petrou et al., 2012). It is also positively related to job performance, organization citizenship behaviors (Buil et al., 2019), and organizational commitment (Uddin et al., 2019), and negatively associated with turnover intention (Jones and Harter, 2005). Workers with high work engagement are more enthusiastic about their job, which imbues energy to the entire organization (Schaufeli et al., 2002). Studies on work engagement have focused mainly on its consequences (Kim et al., 2009).

There is a need to understand the intrapersonal process to work engagement (Inceoglu and Warr, 2011). According to Kahn (1990), an individual’s perception of their work environment and personality traits affect their willingness to engage in work roles. The individual tendency to self or organizational practices represents an important factor in determining how employees adapt to their work and work environments (Salanova and Schaufeli, 2008). Therefore, it is important to examine the relation between personal traits and work engagement to understand the process toward an individual’s career adaptation. In this study, we posited core self-evaluation (CSE) as an intrapersonal factor influencing the work engagement of individuals.

Core self-evaluation is a stable dispositional trait, including a basic assessment of oneself (Judge, 2009; Di Fabio et al., 2012), and provides a framework through which individuals make subjective cognitive appraisal (Judge et al., 1998). CSE is considered as a useful organizing framework that helps to understand individual differences in the coping process (Kammeyer-Mueller et al., 2009). Therefore, it seems to be an adequate variable for examining the internal mechanism of adaptation. CSE consists of the four sub-factors of self-esteem, generalized self-efficacy, locus of control, and emotional stability (Judge et al., 1998). According to Judge et al. (2003), CSE better predicts job and life satisfaction as a single higher-level factor compared with the subset of sub-factors. In studies that examined its relevance to career-related variables, CSE is positively correlated with work engagement (Rich et al., 2010), job satisfaction, and career satisfaction (Stumpp et al., 2010). Further, CSEs positively predict individuals’ career success (Judge and Hurst, 2008).

However, it is important to identify the mechanism of traits influencing work engagement. As interventions for traits are difficult to formulate, it is necessary to check the mediation effect of other possible factors to increase the possibility for interventions. According to Shrout and Bolger (2002), the empirical examination of mediation effects can increase the understanding of the possible points of intervention in counseling. Studies on the relations between work engagement and its antecedents have shown that job and personal resources are positively related with work engagement (Xanthopoulou et al., 2009; Christian et al., 2011; Mazzetti et al., 2016). For these reasons, we posited career adaptability as a personal resource mediating the relation between CSE and work engagement. This is also consistent with career construction theory (CCT; Savickas, 2005), which explains the intrapersonal process of individuals’ adaptation, focusing on career adaptability.

Career construction theory was proposed in response to the increased need for individuals to adjust their own career changes (Savickas, 2005; Niles, 2011). Although previous theories suggest career and personal success can be achieved when the individual and job characteristics match (Dawis and Lofquist, 1984; Holland, 1985), CCT emphasizes individuals’ proactive attitude and behavior for adapting to complex career environments (Tak et al., 2015). In CCT, the process of individuals’ adaptation is explained by a sequential order of adaptivity, adaptability, adapting response, and adaptation (Savickas, 2005). Savickas and Porfeli (2012) defined adaptivity (adaptive readiness) as individuals’ flexibility or willingness to change, whereas adaptability (adaptive resource) is the key component of the CCT model that refers to the individuals’ socio-psychological resources helping them solve unfamiliar, complex, and unclear problem or self-regulate in a rapidly changing environment. Adapting response is the adaptive behavior in a changing environment (Hirschi et al., 2015) through which individuals reach adaptation. In this process, people prepare differently, manage resources differently, and respond differently, resulting in a different degree of integrated attitudes toward the life-span career development process (Savickas, 2012). Therefore, it will be necessary to extend the understanding of these conceptual frameworks and to test the mediational model that includes the relation between individual characteristics and performance (Schmitt and Chan, 2014).

Savickas (2005) asserted that adaptability assumes an environmental change; thus, the actual strategies of adaptability depend on the situation, social roles, and historical time frame. Studies regarding developing and validating the career adaptability scale (CAAR), as well as on the conceptual framework based on the CCT, have been conducted mainly for college students (Savickas and Porfeli, 2012; Yoo and Lee, 2015). Few studies have delved into the integrated process of the career adaptation of working adults. There are differences in strategies related to career adaptability between college students and working adults, given the differences of career development and situations and challenges faced (Tak et al., 2015). The present study intended to integrate the career adaptation process of Korean workers based on CCT.

Career adaptability, the core construct in CCT, is defined as the willingness or capability to cope with uncertainty and unpredictable changes in the career environment by flexibly changing emotions, thoughts, and behaviors (Johnston et al., 2016). Savickas (2005, 2012) constructed career adaptability with 4Cs: concern, control, curiosity, and confidence. Career adaptability can be seen as cumulative competence gained through education and experience (Savickas and Porfeli, 2012). Career adaptability differs from a stable trait in that it is a socio-psychological resource that changes based on the interaction between the individual and the changing environment but also could be influenced by stable trait (Savickas, 1997). Therefore, we posited career adaptability as a mediator in the relation between CSE and work engagement. As such, interventions that increase career adaptability may increase employees’ work engagement.

Savickas (2005) explained that career adaptability is critical for individuals in adapting to the environment and emphasized the process by which individuals construct their own career using career adaptability. Working adults with high career adaptability explore career opportunities with a sense of ease and calmness even in an unpredictable career environment (O’Connell et al., 2008), as well as develop their career with positive thinking and proactive effort (Hirschi et al., 2015). In addition, workers with high career adaptability can rapidly develop their competencies (e.g., knowledge, skills, and abilities) in new environments, which also works toward career success (Pulakos et al., 2000). Thus, career adaptability is important in career development, particularly in the current society marked by rapidly changing environments (Savickas et al., 2009). Studies on career adaptability confirm that high career adaptability is positively related to adaptive outcomes, such as job search behavior, career decision (van Vianen et al., 2012), career planning (Hirschi et al., 2015; Taber and Blankemeyer, 2015), career exploration behaviors (van Vianen et al., 2012; Li et al., 2015), job performance (Ohme and Zacher, 2015), and employability (McArdle et al., 2007), and had a negative relation with turnover intention (Chan and Mai, 2015).

**Hypothesis 1:** CSE of new employees will influence work engagement via career adaptability.

Even if these intrapersonal processes are considered important, the environment that organizations provide in terms of personal career adaptation still needs to be addressed as a major consideration (Cullen et al., 2014). The interaction between the individual and environment should be considered; an individual-centered approach is likewise significant (Lent and Brown, 2019). According to Savickas and Porfeli (2012), career adaptability is related to the person–environment relationship and has a variety of activation states. Therefore, the proper level of work environment should be provided for an individual’s career adaptability to be activated.

Bakker and Demerouti (2007) integrated the influence of job demands and job resources on burnout and work engagement in organization through the Job Demands–Resources model (JD-R model). According to this model, if employees perceive plenty of job demands and the lack of resources to perform these demands on their own, then they experience tension and exhaustion. Bakker and Sanz-Vergel (2013) noted that job demands can weaken the positive relationship between personal resources and work engagement. Thus, we posited that the intra-personal adaptation process will not work properly if the job demands are high enough to make it difficult for individuals to utilize their career adaptability as a personal resource.

Job insecurity is defined as “an overall concern about the continued existence of the job in the future (Sverke et al., 2002, p 243).” In the JD-R model, job demands are defined as work environments and stimuli that require sustained physical and psychological efforts (Bakker et al., 2003). Unstable job conditions have been identified as a major stressor that employees can experience in the workplace (Ironson, 1992) and it require individuals’ effort. Therefore, in many studies, job insecurity has been studied as a job demand (Probst, 2002; Silla et al., 2008; De Cuyper et al., 2009). Studies have also confirmed that job insecurity negatively influences organizational performance and tenure (Cheng and Chan, 2008) and hinders organizational commitment (Roskies and Louis-Guerin, 1990; De Witte and Näswall, 2003). Thus, we posited that excessively high job insecurity will reduce the positive effect of career adaptability on work engagement. In addition, job insecurity is also posited to decrease work engagement by influencing the link between CSE and work engagement.

**Hypothesis 2:** Job insecurity will moderate the effect of new employees’ career adaptability on work engagement.

**Hypothesis 3:** The mediation path from CSE to work engagement via career adaptability will be moderated by job insecurity.

The purpose of this study is to identify the internal process that predicts career adaptability of adults employed within 3 years based on CCT and to identify the role of environmental demands in this process. We posited an internal process of Korean newcomers in which the CSE affects the career adaptability of the career that in turn affects work engagement. In addition, job demand was posited to weaken the path from career adaptability to work engagement, and the indirect path that leads to work engagement. Thus, to test an integrated model, we tested a moderated mediation model. Through this study, the internal mechanism which is very important in the adaptation of Korean worker, but that has not been studied much, can be empirically supported based on the CCT, and the importance of the environment provided by the organization also will be supported. The results of this study will inform both individuals and organizations with hints to develop intervention strategies that increase career adaptability.

## Materials and Methods

### Procedure and Participants

This study is based on newcomers who experience challenges to adapt to work (Saks et al., 2011), therefore a total of 335 Korean full-time employees who had been employed for <3 years (*M* = 1.44 years, *SD* = 0.78 years) participated in the present study. Of the participants, 74.6% (*n* = 250) were women and 25.4% (*n* = 85) were men. In the data analysis, gender was statistically controlled to prevent the effects caused by differences in participants’ gender ratio. The participants reported a mean age of 23.65 years (*SD* = 2.40 years). The occupational field of participants were marketing/sales (*n* = 50, 15.4%), financial accounting (*n* = 50, 15.4%), general affairs/management (*n* = 46, 14.2%), R&D (*n* = 27, 8.3%), HRD (*n* = 17, 5.2%), planning and coordination (*n* = 10, 3.1%), computation development (*n* = 6, 1.9%), and others (*n* = 85, 26.2%). They were invited through the online data collection service Data Spring. The online survey took approximately 30 min to complete; participants who completed the survey received 500 points give in return. The Institutional Review Board of Yonsei University approved the procedure and contents of the study. We explained the purpose of the study and reassured participants that participation was voluntary, and the data collected would remain confidential. To ensure the quality and reliability of data, data were screened for outliers. The standardized residuals were used to detect outliers and excluded from the analysis when absolute value was >2 (Hoeting et al., 1996). Finally, data from 324 participants (women = 242; 74.7%) were included in the analysis.

### Measures

#### CSE

Judge et al.’s (2003) CSE scale translated by Tak (2007) was used. The CSE scale is a 12-item measure; example items include “I am confident I get the success I deserve in life” and “When I try, I generally succeed.” Each item is rated on a five-point Likert-type scale ranging from 1 (*strongly disagree*) to 5 (*strongly agree*). The scale scores are the sum of the ratings of the items. Relevant items were reverse-coded. In the present study, the internal consistency was 0.79.

#### Career Adaptability

Career adaptability was assessed using the Korean version of the CAAS (Tak et al., 2015) developed by Savickas (2012) based on samples of employees. The international CAAS is a 24-item measure assessing four dimensions of career adaptability: concern, control, curiosity, and confidence. Tak et al. (2015) found that a four-factor model with 16 items of CAAS is appropriate for Korean employees. Therefore, we used the 16-item measure in the present study. Example items include “Becoming aware of the educational and vocational choices that I must make” and “Taking responsibility for my actions.” Participants answered using a five-point Likert scale ranging from 1 (*not strong*) to 5 (*strongest*), with higher scores indicating higher levels of career adaptability resource. The Cronbach’s alpha for the scale in the present study was 0.91.

#### Job Insecurity

The assessment of job insecurity was based on Ashford et al.’s (1989) Job Insecurity scale, which measures perceived threats to the job. Kim (2015) translated and reconstructed this scale, and we used Kim’s (2015) scale. The nine-item scale measures such items as “I may lose my job and be moved to a lower level within the organization” and “I am likely to be pressured to accept early retirement.” Participants answered items on a five-point Likert scale ranging from 1 (*strongly disagree*) to 5 (*strongly agree*). In the present study, internal consistency reliability of the scale was 0.83.

#### Work Engagement

To assess employees’ work engagement, we used the Work Engagement scale (Schaufeli et al., 2006) translated and reconstructed by Hwang (2017). The scale has 10 items however we decided to use only three items to analysis not to undermine the meaning of original scale. The items are “At my work, I feel bursting with energy” (vigor), “I am enthusiastic about my job” (dedication), “I am immersed in my work” (absorption). Participants answered items on a five-point Likert scale ranging from 1 (*almost never*) to 5 (a*lways*). In the present study, the Cronbach’s alpha for the scale was 0.72.

### Data Analysis

The present study was designed to test a moderated mediation hypothesis. Moderated mediation means the mediation effect changes depending on the level of the moderator variable (Mueller et al., 2005). The present research model hypothesized the mediating relation of CSE → career adaptability → work engagement and that job insecurity moderated the mediation process of CSE, career adaptability, and work engagement. We used the SPSS PROCESS Macro suggested by Hayes (2012) to test the hypotheses. According to Mueller et al. (2005), establishing moderated mediation requires estimating parameters for three statistical models. First, we conducted multiple regression analysis using SPSS PROCESS Macro model 1 and then tested the total direct effect of CSE on work engagement, which was not moderated by job insecurity. The prototypic case of moderated mediation showed a total direct effect, and the magnitude of this effect did not change depending on the moderator (Mueller et al., 2005). Second, multiple regression was conducted on the indirect effect without the moderator; bootstrapped confidence interval (5000 bootstrap samples) was obtained using SPSS PROCESS Macro Model 4. Finally, to assess the complete moderated mediation model, Model 14 was specified in the SPSS PROCESS Macro. We noted imbalances in gender. The demographic factor could influence many job-related variables. Therefore, we included these variables as controls in our hypothesis test.

## Results

Table 1 presents the descriptive statistics and correlations for the study variables. CSE correlated strongly with career adaptability (*r* = 0.52, *p* < 0.01) and work engagement (*r* = 0.38, *p* < 0.01), and moderately with job insecurity (*r* = −0.31, *p* < 0.01). Career adaptability correlated slightly with job insecurity (*r* = −0.12, *p* < 0.05) and moderately with work engagement (*r* = 0.32, *p* < 0.01). Job insecurity correlated slightly with work engagement (*r* = 0.13, *p* < 0.05).

**TABLE 1:** Descriptive statistics and correlations of study variables.

**Variables**	***M***	***SD***	**1**	**2**	**3**	**4**	**5**
1. Gender	0.75	0.44	–				
2. CSE	37.51	5.87	–0.19^∗∗^	–			
3. Career adaptability	58.40	8.26	−0.14^∗^	0.52^∗∗^	–		
4. Job insecurity	23.64	5.87	–0.15^∗∗^	–0.31^∗∗^	−0.12^∗^	–	
5. Work engagement	8.27	2.47	–0.25^∗∗^	0.38^∗∗^	0.32^∗∗^	0.13^∗^	–

**N* = 324, ^∗∗^*p* < 0.01, ^∗^*p* < 0.05. CSE, core self-evaluation.*

### Hypothesis Test

**FIGURE 1:**
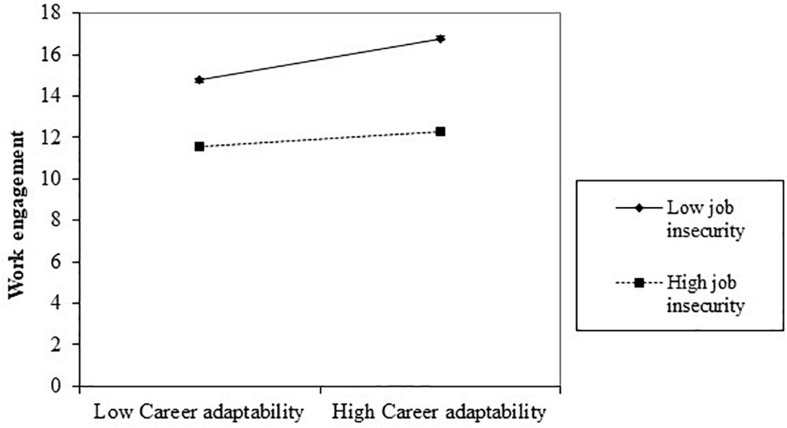
The moderation effect of job insecurity on Career adaptability to work engagement.

We expected that job insecurity would moderate the indirect association between CSE and work engagement via career adaptability. To test this moderated mediation hypotheses, we used the integration approach suggested by Mueller et al. (2005). This approach includes three regression models: moderation, mediation, and moderated mediation. In the analyses, variables were centered, and control variables were set in the first block (Aiken et al., 1991). The specification of these models can be seen in Table 2. First, we tested the total direct effect of CSE on work engagement; the output indicated an insignificant interaction effect of CSE and job insecurity on work engagement (β = −0.01, *p* = 0.79, see Table 2). Thus, the magnitude of the total direct effect did not change according to job insecurity. Second, we conducted the multiple regression analysis and tested the mediation effect without the moderator; it showed a partial indirect effect of CSE on work engagement via career adaptability as hypothesized (Δ*R*^2^ = 0.02, *p* < 0.01). Third, we tested the significance of the indirect effect using bootstrapping technique (Shrout and Bolger, 2002); the bootstrapped confidence interval [95% CI: (0.01, 0.06)] did not include zero. Thus, the indirect effect was significant. Finally, we tested the moderated mediation model using SPSS PROCESS Macro Model 14. Of the analysis on the moderator variable, career adaptability was found to interact with the moderator, job insecurity, on the dependent variable, work engagement (β = −0.10, *p* < 0.05). Thus, the effect of career adaptability on work engagement varied depending on the level of job insecurity (see Figure 1).

**TABLE 2:** Results for testing hypotheses.

	**β**	***SE***	***t***	**LLCI**	**ULCI**	***R*^2^**	***F***

**Moderation analysis (moderation effect of *X*–*Y*)**							
**Outcome variable: work engagement**							
Constant	–	0.26	34.56^∗∗∗^	8.31	9.32	0.22	22.89^∗∗∗^
Gender	–1.82	0.29	−2.61^∗^	–1.34	–0.19		
CSE	0.41	0.02	7.41^∗∗∗^	0.13	0.22		
Job insecurity	0.24	0.02	4.42^∗∗∗^	0.06	0.14		
CSE × job insecurity	–0.01	0.00	–0.65	–0.01	0.00		

**Mediation analysis**							

**Outcome variable: career adaptability**							
Constant	–	2.80	11.37^∗∗∗^	26.34	37.36		
Gender	–0.04	0.92	–0.86	–2.60	1.02		
CSE	0.51	0.07	10.61^∗∗∗^	0.59	0.86		
**Outcome variable: work engagement**						0.27	60.74^∗∗∗^
Constant	–	1.05	2.11^∗^	0.15	4.28		
Gender	–0.18	0.29	–3.54^∗∗∗^	–1.60	–0.46		
CSE	0.26	0.03	4.36^∗∗∗^	0.01	0.16		
Career adaptability	0.16	0.02	2.64^∗∗^	0.01	0.08		

**Moderated mediation analysis**							

**Outcome variable: career adaptability**							
Constant	–	2.80	–9.48^∗∗∗^	–32.06	–21.04	0.27	60.74^∗∗∗^
Gender	–0.04	0.92	–0.86	–2.60	1.02		
CSE	0.51	0.07	10.61^∗∗∗^	0.59	0.86		
**Outcome variable: work engagement**							
Constant	–	1.04	3.45^∗∗∗^	1.54	5.63	0.25	20.92^∗∗∗^
Gender	–0.13	0.29	−2.57^∗^	–1.31	–0.17		
CSE	0.33	0.03	5.38^∗∗∗^	0.09	0.19		
Career adaptability	0.16	0.02	2.49^∗^	0.01	0.08		
Job insecurity	0.24	0.02	4.63^∗∗∗^	0.06	0.15		
CA × Job insecurity	–0.10	0.00	−2.09^∗^	–0.01	–0.00		

**N* = 324. CA, career adaptability; CSE, core self-evaluation; LL, low limit; CI, confidence interval; UL, upper limit. Gender was dummy coded. Bootstrap sample size = 5000. ^∗^*p* < 0.05, ^∗∗^*p* < 0.01, ^∗∗∗^*p* < 0.001.*

Regarding the total moderated mediation effect, the index of moderated mediation (Hayes, 2015) is displayed in Table 3. The effect was significant [95% CI: (−0.01, −0.00)], indicating that the indirect effect of CSE on work engagement through career adaptability was moderated by job insecurity.

**TABLE 3:** Index of moderated mediation.

	**Index**	**Boot SE**	**Boot LLCI**	**Boot ULCI**
Job insecurity	–0.0038	0.0018	–0.0074	–0.0002

*Bootstrap size = 5000, bootstrap confidence interval = 95%. LL, low limit; CI, confidence interval; UL, upper limit.*

**FIGURE 2:**
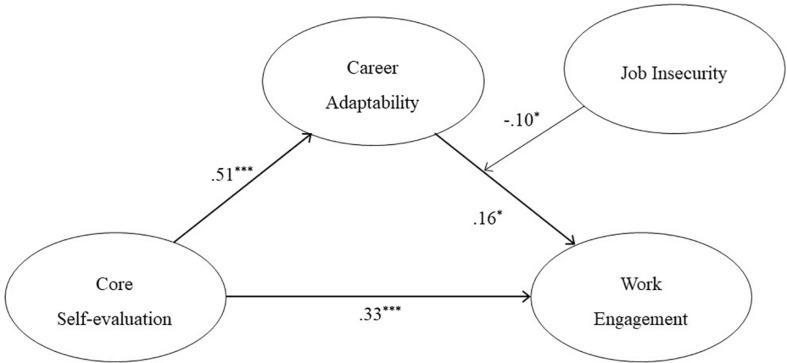
Theoretical research model with standard coefficients.

The conditional indirect effect on values of the moderator was calculated: the mean, one standard deviation above (+5.87), and one standard deviation below (−5.87). The results are shown in Table 4. The indirect effect was significant for both low job insecurity [95% CI: (0.02, 0.09)] and the mean [95% CI: (0.01, 0.05)]. However, the indirect effect was not significant for high job insecurity [95% CI: (−0.02, 0.04)]. Thus, the indirect effect of CSE on work engagement via career adaptability could not be achieved in a high job insecurity environment. To activate workers’ positive inter-personal process for work engagement, employers should provide a good enough environment (see Figure 2).

**TABLE 4:** Results for conditional indirect effect analysis.

**Job insecurity**	**Effect**	**Boot SE**	**Boot LLCI**	**Boot ULCI**
−1 *SD* (−5.87)	0.05	0.02	0.02	0.09
Mean	0.03	0.01	0.01	0.05
+1 *SD* (+5.87)	0.01	0.02	–0.02	0.04

*Bootstrap size = 5000. *SD*, standard deviation; LL, low limit; CI, confidence interval; UL, upper limit.*

## Discussion

This study explored the internal mechanism that influences a new employee’s career adaptation and identified the work environment that could facilitate this mechanism among Korean newcomers. Based on CCT, we examined the mediation effect of career adaptability on the relation between CSE and work engagement and the moderating role of job insecurity as a job demand in the mediation process. We hypothesized mediation effect of career adaptability between CSE and work engagement, moderation effect of job insecurity on the relationship between new employees’ career adaptability and work engagement, and comprehensively we expected that the hypothesized mediation path would be moderated by job insecurity All the hypothesis was supported. The results showed that as CSE, a fundamental trait, strengthens, adaptability increases, which in turn leads to workers feeling more work engagement. The results also showed that job insecurity moderated this internal adaptation process as a job demand that requires psychological and physical effort.

In other words, a person with a higher CSE level has a higher level of career adaptability, which leads to better work engagement. However, if the person perceives a higher level of job insecurity, then the positive effects of career adaptability on work engagement would be lower. In addition, an individual internal adaptation process would not work in a group with a high job insecurity of +1 *SD*, suggesting a potent environmental impact on the adaptation of new employees. According to Conservation of Resources theory (COR theory; Hobfoll, 1989), individuals have an intrinsic desire to acquire, conserve, and protect resources they have. As a result, individuals seek to conserve and invest resources to anticipate future losses, and burnout when they feel threatened or actually lost, and when they invest resources but do not get enough compensation (Hobfoll, 2001). Therefore, the result of this study is consistent with the theoretical background of COR theory in that it has been found that the impact on work engagement is lowered when the level of job insecurity that threatens career adaptability as an individual resource exceed the range that an individual can afford.

According to the JD-R model (Bakker and Demerouti, 2007), when individuals feel that they are facing plenty of job demands and lacking the resources to do so, they show low engagement for their jobs and high burnout. Career adaptability, meanwhile, is defined as an in-person resource that allows individuals to adapt to the needs of the environment (Savickas and Porfeli, 2012). These results suggested that career adaptability resources are also likely to fail to work in the adaptation process in a highly demanding situation. The results of this study contribute to the expansion of the CCT in identifying the effect of environment in the internal mechanism of Korean newcomers.

Specifically, we found support for the partial indirect path from CSE to work engagement via career adaptability of employees who had been employed for <3 years. The result revealed that career adaptability is the factor that links the relation between positive personal trait (CSE) and work engagement. This result is also consistent with the sequential relation of adaptivity, adaptability, adapting response, and adaption of CCT (Savickas and Porfeli, 2012). In Korea, new recruits enter the workforce after a highly competitive job search process, yet they are asked to take care of their own careers (Kim and Jyung, 2011). The contribution of this study is that CCT was empirically tested in a sample of Korean newcomers. The average working hours of Korean workers is 8.3 h per day, which is longer compared to the average working hours of 6.6 h per day in OECD countries. Therefore, for Korean employees who spent most of their day at work, successful career adaptation should be especially important. Many of them fail to do so, being unable to adapt to their work; indeed, the rate of those quitting is rising (The Korea Employers Federation [KEF], 2016). In a 2017 survey, the percentage of employees who leave the company within 3 years reached a striking 62.2% (Hyun, 2017). This indicator of individual failures can lead to personal and organizational losses. Thus, it is important to recruit employees with high CSE, and also implies the need for intervention to increase personal resources. Therefore, research should be conducted to identify the adaptation process and ways to intervene. This study is meaningful in that it supports the application of CCT to new employees in Korea and opens up potential areas of future intervention.

In addition, we tested CSE as an adaptivity variable in CCT and found that it affected career adaptability and work engagement, in line with previous studies that showed a positive correlation between CSE and career adaptability (Zacher, 2014a, b; Hirschi et al., 2015). Our results further supported previous findings that CSE makes individual more ambitious and confident in their career, thereby making employees actively engaged in career planning and job-related behaviors (Judge et al., 1997). CSE is considered a fundamental trait with a broader conceptual range compared with the existing dispositional traits (Judge and Bono, 2001). The fact that this study supports the role of CSE as an adaptivity variable of CCT suggests that the results provide a broader and more comprehensive framework for understanding adaptability compared with previous links between personality traits, career-related attitudes, and behavioral variables (Johnson et al., 2008).

The results of this study can be used to provide practical intervention for the adaptation of employees. New employees in Korea mainly quit their jobs because they fail to adapt to the organization or job role (The Korea Employers Federation [KEF], 2016). This scenario suggests the need for active intervention on new employees’ adaptation to prevent great losses for both individuals and organizations. Our results showed that individuals with positive self-evaluation tend to have a higher level of career adaptability resources, and consequently, feel more enthusiastic about their job. In addition, if the job insecurity is high, then the positive internal process does not work. Thus, organizations should be aware of the importance of providing training that strengthens career adaptability and an environment that is stable and secure, as well as of putting effort in recruiting individuals with high CSE.

The limitations of this study and implications for future research are as follows. First, the proportion of women among the participants was overly high. We statistically controlled the influence of gender, but there may be limitations in generalizing the findings. As this study used self-report questionnaires, there was a possibility that the participants gave biased responses, such as socially desirable responses. Second, although the study included the environmental constraints that weaken individual adaptive processes, it did not include the environmental resources that could increase adaptability. Thus, it is necessary to explore the effect of both job resources and demands in future studies as job resources may help individuals overcome job demands (Bakker et al., 2005). Third, this study has a limitation that it is difficult to generalize because the number of participants if limited. Lastly, the cross-sectional study design only tested the influence of CSE of new employees on work engagement through career adaptability. The cross-sectional design has a limitation because the CSE and work engagement are simultaneously assessed there is generally no evidence of a temporal relationship between CSE and work engagement. We suggest that this relation be tested in a longitudinal study in future.

## Data Availability

The datasets generated for this study are available on request to the corresponding author.

## Ethics Statement

This study was carried out in accordance with the recommendations of the Yonsei University Institutional Review Board. The protocol was approved by the Yonsei University Institutional Review Board. Participants gave written informed consent in accordance with the Declaration of Helsinki.

## Author Contributions

KY designed the study, collected the data, and performed the data analysis under the supervision of K-HL. K-HL provided the critical revisions. Both authors approved this work and the final version of the manuscript for submission.

## Conflict of Interest Statement

The authors declare that the research was conducted in the absence of any commercial or financial relationships that could be construed as a potential conflict of interest.
